# Validity and variability of xBRS: instantaneous cardiac baroreflex sensitivity

**DOI:** 10.14814/phy2.13509

**Published:** 2017-11-28

**Authors:** Karel H. Wesseling, John M. Karemaker, Paolo Castiglioni, Emil Toader, Andrei Cividjian, Jos J. Settels, Luc Quintin, Berend E. Westerhof

**Affiliations:** ^1^ BMEye Amsterdam The Netherlands; ^2^ Department of Medical Biology (Section Systems Physiology) Academic Medical Center University of Amsterdam Amsterdam The Netherlands; ^3^ IRCCS Don C. Gnocchi Foundation Milan Italy; ^4^ Department of Physiology University of Lyon Lyon France; ^5^ Edwards Lifesciences BMEYE Amsterdam The Netherlands; ^6^ Department of Pulmonary Diseases VU University Medical Center Amsterdam The Netherlands

**Keywords:** Atropine, autonomic variability, cardiac autonomic blockade, cardiac baroreflex, cardiac–respiratory interaction, clonidine, cross‐correlation baroreflex, propranolol, xBRS

## Abstract

Spontaneous oscillations of blood pressure (BP) and interbeat interval (IBI) may reveal important information on the underlying baroreflex control and regulation of BP. We evaluated the method of continuously measured instantaneous baroreflex sensitivity by cross correlation (xBRS) validating its mean value against the gold standard of phenylephrine (Phe) and nitroprusside (SNP) bolus injections, and focusing on its spontaneous changes quantified as variability around the mean. For this purpose, we analyzed data from an earlier study of eight healthy males (aged 25–46 years) who had received Phe and SNP in conditions of baseline and autonomic blocking agents: atropine, propranolol, and clonidine. Average xBRS corresponds well to Phe/SNP‐BRS, with xBRS levels ranging from 1.2 (atropine) to 102 msec/mmHg (subject asleep under clonidine). Time shifts from BP‐ to IBI‐signal increased from ≤1 sec (maximum correlations within the current heartbeat) to 3–5 sec (under atropine). Plotted on a logarithmic vertical scale, xBRS values show 40% variability (defined as SD/mean) over the whole range in the various conditions, except twice when the subjects had fallen asleep and it dropped to 20%. The xBRS oscillates at frequencies of 0.1 Hz and lower, dominant between 0.02–0.05 Hz. Although xBRS is the result of IBI/BP‐changes, no linear coherence was found in the cross‐spectra of the xBRS‐signal and IBI or BP. We speculate that the level of variability in the xBRS‐signal may act as a probe into the central nervous condition, as evidenced in the two subjects who fell asleep with high xBRS and only 20% of relative variation.

## Introduction

The sensitivity of the arterial baroreceptor‐heart rate reflex or cardiac baroreflex sensitivity (BRS) is calculated from the ratio of the heart period changes subsequent to systolic blood pressure changes in msec/mmHg. In a previous paper (Westerhof et al. 2004), we described a method for BRS computation from spontaneous cardiovascular oscillations, using a cross‐correlation technique over a 10 sec wide sliding window (**xBRS**‐technique, see Methods). We showed that the time‐averaged xBRS values are close to those of the Eurobavar data set which we compared it to, but with a larger number of measurements of BRS per minute. A method with a high time‐resolution is useful to uncover and follow instantaneous variations in BRS. In further exploration, we found the standard deviation of the xBRS values to be proportional to its mean value. In other words, variability defined as the coefficient of variation (SD/mean) was almost constant, irrespective of the mean level of BRS. By repeating the computations on reshuffled data sets, we established that this is not due to the computational method. Are the produced numbers a good representation of baroreflex activity? We sought to establish this by teaming up with Dr. Quintin and his group who had published on a BRS‐study (Parlow et al. 1995) with eight healthy subjects. They had used phenylephrine (Phe) and nitroprusside (SNP) bolus injections to determine the gold standard of BRS‐measurements (Smyth et al. 1969) and the “tangent method” or **TG‐BRS** (Parlow et al. 1995) reference value from the combination of those two. Moreover, the injections had been repeated under autonomic blockade with atropine, propranolol and clonidine, whereby different heart rates and different vagal/sympathetic effector balances had been established. We applied xBRS to this data set to assess xBRS for accuracy and its apparent reflex delay in establishing “true” baroreflex sensitivity, while at the same time assessing the variability of the values under various forms of autonomic blockade. It was predicted that the time‐averaged values of xBRS would follow the Phe/SNP BRS‐results, that the variance of xBRS would change in relation to its mean level and that reflex delay would increase from 0 to 1 sec at normal resting heart rates to 3–4 sec under atropine and it would decrease to the shortest values at longer resting intervals, under propranolol and clonidine (Cividjian et al. 2011). Finally, we applied a Fourier transform to the sequentially measured BRS‐values to unmask possibly recurrent oscillations in an ostensibly random signal, comparing this to the underlying interbeat interval – and blood pressure signals by cross‐spectral analysis.

## Methods

### Subjects

For this study, the recordings from the previous study by Parlow et al. (1995) were reanalyzed. In brief, having obtained approval from the ethics committee of the Hospices Civils de Lyon and signed informed consent of the volunteers, eight healthy, normotensive, male physicians, aged 25–46 years, of average height and build were studied. All were in good physical shape, some were long‐distance runners (and had, consequently, low resting heart rates). They were instructed to avoid tobacco, alcohol, and caffeine for 12 h and strenuous exercise for 24 h before the measurements. Subjects were coded randomly from the set [A…Z].

### Protocol and measurements

The protocol has been described in detail before (Parlow et al. 1995). In short, three sessions were scheduled for each subject in the morning of 3 days separated by approximately 2 weeks. Recordings were performed with the subjects in the supine position, in a hospital bed, in a dimly lit room. After a baseline recording each day, the first day atropine sulfate (40 μg/kg·i.v.) was administered as a bolus, followed by a reinforcing dose of atropine (10 μg/kg·i.v.) plus propranolol (200 μg/kg·i.v. over 5 min). On the second day the baseline recording was followed by a study period with propranolol (200 μg/kg·i.v.). On the third day, the baseline recording was followed by a study period with clonidine hydrochloride (6 μg/kg taken orally) and the recording was made after 2 h had elapsed. In each condition, a 20‐min stable period was recorded which we used to establish spontaneous xBRS, followed by a short break and a period in which several injections of Phe and SNP were given to establish drug‐induced BRS. The various conditions were studied separately and are called study periods: **b1, b2,** and **b3** for baselines of 3 days, **a** for atropine, **p** for propranolol, **c** for clonidine. Continuous blood pressure was monitored with a noninvasive Finapres 2300 (Ohmeda, Englewood, USA) which gave beat to beat systolic pressure and interbeat interval data.

### Analysis

Spontaneous baroreflex sensitivity was assessed with the xBRS method detailed earlier (Westerhof et al. 2004) Briefly, a 10 sec window progresses in one‐second steps over the pressure and interbeat interval records and the signals within the window are cubic spline interpolated and resampled at a 1 sec rate. Next, pressure and interval are cross‐correlated with 0, 1, 2…5 sec delay compensation for interval and the delay with the highest cross‐correlation is taken as optimal delay *τ*. If the highest cross‐correlation is positive and significant at *P* ≤ 0.05, the ratio of the standard deviation of interbeat interval against that of pressure is filed as one determination of xBRS. Thus, the ratio is taken of the interbeat interval variability (msec) to the systolic pressure variability (mmHg) as BRS‐number only when a significant degree of correlation is present. This is mathematically equivalent to, but computationally more efficient than dividing the regression coefficient by the correlation coefficient as we did in the previous study (Westerhof et al. 2004). In an earlier study, we used a probability level *P* ≤ 0.01 for significance. The higher *P*‐level of 0.05 almost doubles the number of determinations per minute without an appreciable change in the mean BRS value or its standard deviation (Westerhof et al. 2006). Except for the *P*‐level, the xBRS method requires no thresholds, not for pressure‐ or for interval changes (this issue is discussed in more detail in the Appendix). The optimal delay is determined instead of assuming a fixed delay, as in some other spontaneous sequences methods (Di Rienzo et al. 1985). When the end of the input file is met, the geometric mean and ‐ standard deviation of all determinations of xBRS in the output file are computed and the distribution of optimal delays *τ* is assembled to be used for further analysis.

To compute the cross‐spectrum of beat‐to‐beat IBI or systolic pressures with xBRS, we wrote a special‐purpose Matlab^®^ program. This computed xBRS‐values as described and assigned each value that fulfilled the criteria to the middle heartbeat of the 10‐sec window. Missing values were interpolated; these files were analyzed with Matlab^®^ DFT. We adopted the nomenclature proposed by the TaskForce (1996) for the various frequency bands: ultralow frequencies (ULF): 0–0.003 Hz; very low frequencies (VLF): 0.003–0.04 Hz; low frequencies (LF): 0.04–0.15 Hz and high frequencies (HF): 0.15–0.4 Hz.

From the Phe/SNP‐estimates, reference BRS was obtained with the “tangent to sigmoid method” published earlier (**TG‐BRS** (Parlow et al. 1995)). Rescaled nitroprusside (SNP) – phenylephrine (Phe) response data allow the generation of a sigmoidal cardiac baroreflex curve. The tangent to this curve at resting pressure gives the slope to be expected for xBRS. The BRS estimated from the Phe and SNP responses were also computed individually, using linear regression. The TG BRS technique reduces the positive bias resulting from taking only Phe responses (fast cardiac parasympathetic activation) versus the negative bias from only SNP responses (sluggish sympathetic activation and parasympathetic withdrawal) as reference techniques (Parlow et al. 1995).

### Statistics

The geometric mean of the xBRS values per study period of 20 min was used as it is a better estimate of central tendency when the distribution of the values is skewed and approximately log‐normal. Since BRS is the ratio of the two normal distributions of interval and pressure, the distribution of their ratio is log‐normal. The geometric mean and the standard arithmetic mean produce the same numbers on normal distributions, thus geometric averaging is applicable to both distributions. The geometric mean (gM) and geometric standard deviation (gSD) were obtained over each 20 min study period. This was done by first taking the natural logarithm (log_e_ or ln) of the xBRS values (by definition always positive), by computing mean and standard deviation of the log‐transformed xBRS; and by presenting the values back in msec/mmHg after exponential transform (e^*x*^).^1^ The geometric mean is less sensitive to the presence of an occasional outlier value. Values for a group of subjects were averaged using the ordinary mean and standard deviation, as these distributions appear normal. Plotting xBRS data on a logarithmic scale, in view of the high degree of variability, has the advantage that at very low and very high xBRS levels variability can be observed with the same relative (or percentage) sensitivity.

The xBRS distributions were tested with the Kolmogorov–Smirnoff test, separately for the eight subjects and seven study periods (baseline or drug) to establish whether the distribution types classified as normal or log‐normal. Nonparametric statistical tests on the data were performed when required. Thus, Wilcoxon for matched pairs testing, Spearman rank correlation and Friedman two‐way ANOVA were used, as indicated in the text. The distributions of optimal delay τ were compared with a *χ* ^2^ test. Comparing distributions of *τ* in this way is a much more sensitive test for differences in delay than just comparing their means.

## Results

### Volunteer statistics

Baseline (b) systolic pressures and interbeat intervals averaged over three sessions for the eight subjects are presented in Table 1.

**Table 1:** Average baseline (b) systolic pressure (mmHg) and interbeat interval (msec) for the eight volunteer subjects on day 1

Subject	Systolic pressure	Interbeat interval
Code	mmHg	msec
A	111	1006
F	109	893
J	120	1227
P	127	1100
Q	104	1464
R	100	1054
V	121	916
Z	117	960

### Comparison with gold standard drug‐induced and tangent methods

**Figure 1:**
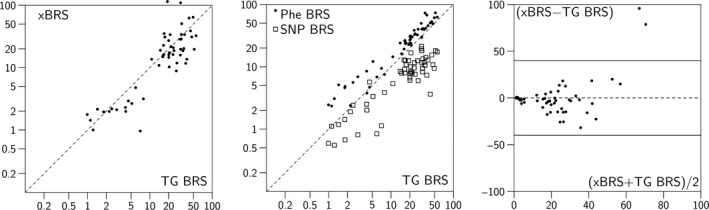
From left to right: xBRS versus the TG BRS method; Phe and SNP BRS versus TG BRS (note the log‐log scales); Bland and Altman plot of the difference between xBRS and TG BRS sensitivities versus their average (linear scales). The Phe BRS method produces higher and the SNP BRS method lower values than TG‐BRS. The xBRS produces BRS values on average identical to the TG BRS method. Scatter is substantial, lowest in the Phe BRS method. All correlation coefficients are significant at *P* = 0.0005. The Phe and TG BRS methods follow each other rather closely but with a substantial offset.

**Figure 2:**
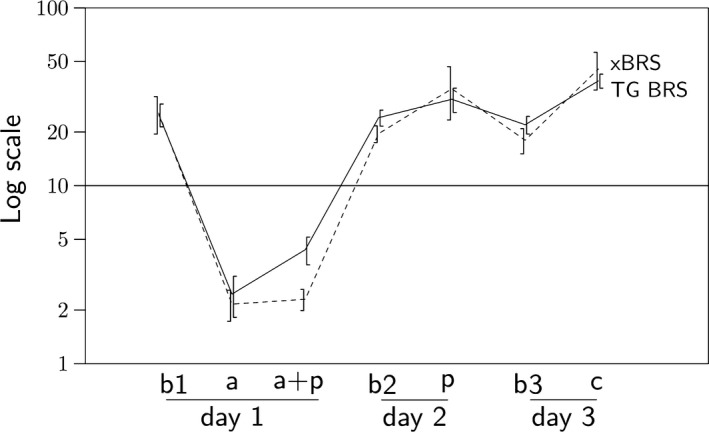
The course of BRS (msec/mmHg) averaged over the population as a function of baseline and drug states. Drawn line is the TG BRS method, dashed line is xBRS. The error bars are ±SEM. The letters **b1**,** b2**, and **b3** denote baseline recordings on days one, two and three, **a** denotes atropine, **a + p** is atropine plus propranolol, **p** is propranolol, and **c** denotes clonidine. Only drug state **a + p** shows a significant difference (factor of 2) between xBRS values and the TG BRS reference. Note the logarithmic vertical scale.

The individual Phe and SNP response results, the TG and xBRS are compared in various ways in Figure 1. Averages over the eight subjects in Figure 2 show the changes in xBRS and tangent baroreflex sensitivities versus the study periods. Note that xBRS values track the tangent values well. Only for atropine + propranolol, a factor of 2 discrepancy was apparent, but results under “total” combined blockade are doubtful anyway, as will be discussed later.

Table 2 lists the xBRS averages per study period. The differences between the three baseline value sets were not significant for either method, TG BRS or xBRS. The three baseline sets per method, however, were uncorrelated. Hence, it is impossible to predict the second or the third baseline value for a subject on day two or three from the first. Atropine decreased TG BRS on average by a factor of 10 (Figure 2). The strength of the effect of atropine is variable per subject ranging from a factor 5 to 27 (5–34 for xBRS). The addition of propranolol caused a 75% increase in TG BRS compared to atropine alone, which was not reflected in xBRS. Compared to their corresponding baseline values, propranolol and clonidine increased TG BRS on average by a factor 1.3 and 1.8, respectively (1.8 and 2.5 with xBRS).

**Table 2:** Baroreflex sensitivity BRS (msec/mmHg) per subject state (baseline or drug) obtained with the xBRS method, each state averaged over the eight subjects

Condition	b1	a	ap	b2	p	b3	c
xBRS	25.6	2.16	2.30	19.6	35.1	18.0	45.4
SEM	6.1	0.43	0.31	2.1	11.7	2.9	10.9
N	30	19	28	30	27	31	33
τ	1.10	2.99	2.62	1.07	0.98	1.33	0.94

Top line presents condition: b1, baseline day 1; a, atropine; ap, atropine plus propranolol; b2, baseline day 2; p, propranolol; b3, baseline day 3; c, clonidine. N, number of xBRS determinations per minute. *τ*, optimal delay of xBRS in seconds.

A two‐way Friedman nonparametric ANOVA performed in both directions on the 56 study period averages indicated significant differences in xBRS between study periods and less significant differences between subjects. Significant at *P* < 0.05 were the differences between study period atropine + propranolol and the others and between atropine and the others. Between atropine and atropine + propranolol the difference was not significant.

### BRS variability

**Figure 3:**
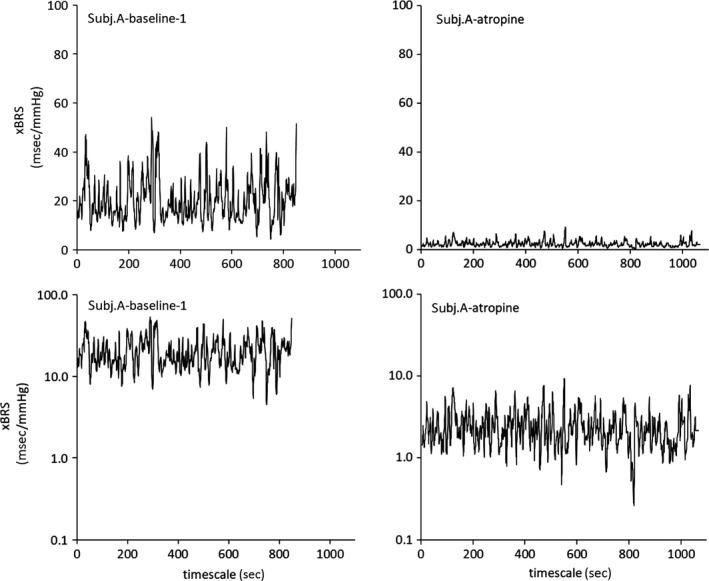
A typical example of the individual xBRS (msec/mmHg) values plotted versus time in seconds. Upper panels on a linear, lower panels on a logarithmic vertical scale. Left panels baseline recording, right panels after atropine administration. Although xBRS level and variability are strongly reduced by atropine, the logarithmic plot shows that variability is proportionally similar. In a previous publication (Karemaker and Wesseling 2008), a preliminary version of this figure has been shown.

A key finding of the xBRS method is shown in Figure 3. It demonstrates in one typical subject (code A) that xBRS fluctuates around a constant level during each study period of which only two are shown. Administration of atropine shortened heart period and lowered xBRS, in this case by a factor of 11, but did not reduce it to zero. Clearly, its variability was also reduced. The two bottom panels display the same data but plotted on a logarithmic vertical scale. On this logarithmic scale, the variabilities in the xBRS values are similar in amplitude. This suggests that the xBRS level had shifted to a lower mean level but its scatter remained proportional to that mean level.

**Figure 4:**
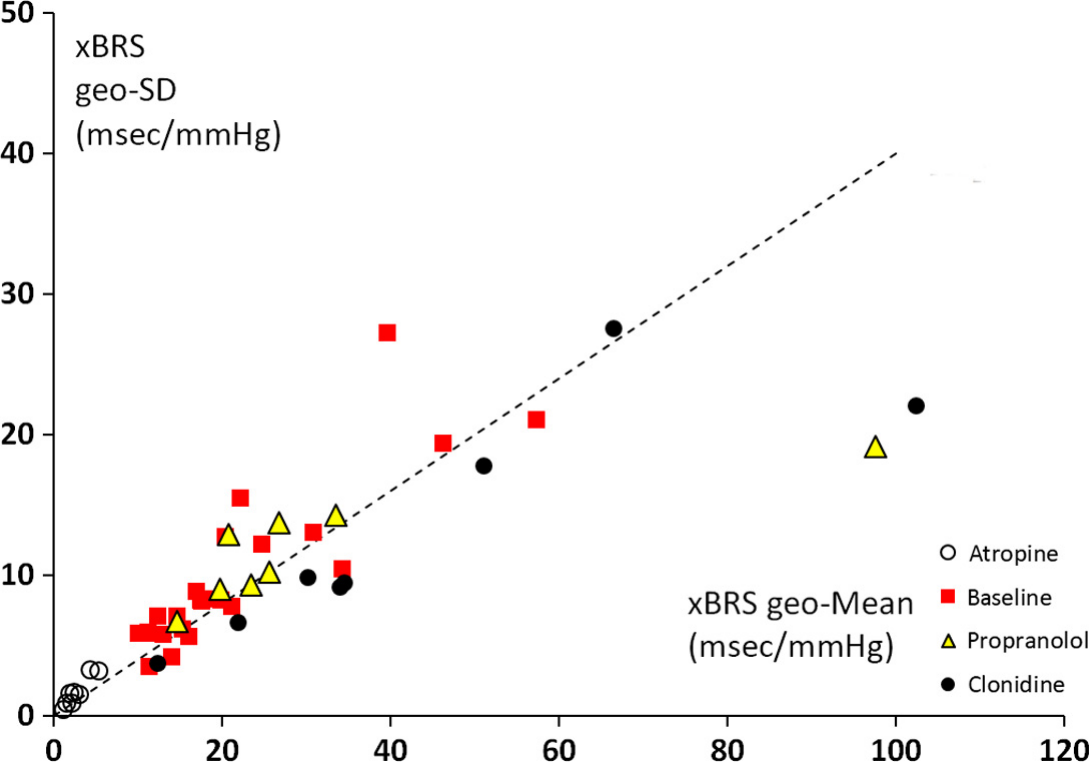
xBRS geometric standard deviation (geo‐SD) is plotted versus geometric mean (geo‐Mean) for all subjects and four different conditions (cf embedded legends), showing proportionality between both parameters. Note that geo‐SD/geo‐Mean is around 0.4 for all values but two outliers to the far right (see text). On these occasions, the subjects had fallen asleep, one after propranolol and one after clonidine administration. Both had the highest xBRS values (around 100 msec/mmHg) and much lower than 0.4 relative standard deviation (geo‐SD/geo‐Mean).

Similar geometric standard deviation to mean ratios (gSD/gM) were computed for all subjects and study periods and the 48 results are plotted in Figure 4. Note that the data points fall on or near a +0.4 slope, with two exceptions: in both cases, the test subject had fallen asleep with very low heart rates. This finding is not an artifact since there are no peculiarities in the recording and computationally the algorithm can reach any number. Possibly, it has to do with the condition of sleep per se.

### Spectral analysis

**Figure 5:**
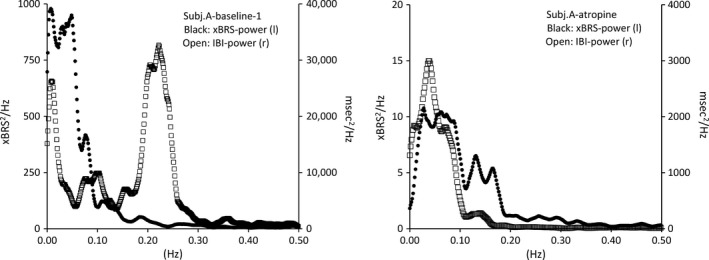
Power spectra of xBRS (round, black) and interbeat interval (IBI, open squares); left baseline recording on day 1, right after atropine. The time curves of xBRS are shown in Figure 1. Under atropine, the power in xBRS is about two orders of magnitude lower than in baseline (note the adapted scales) and there is no respiratory peak to be detected in the IBI‐spectrum.

The xBRS, when plotted as time curve, (Fig. 3), does not make the impression of a random process. Rather some slow oscillations, in the range of 0.02–0.05 Hz, do appear under the various conditions in each subject, be it not as a constant feature and not always at the precise same frequencies. This was apparent in the frequency spectra, which are shown in Figure 5, for the signals of Figure 3. Very low frequency (VLF) peaks are present around 0.03 Hz, confirming the visual impression.

### Optimal delay *τ*

**Figure 6:**
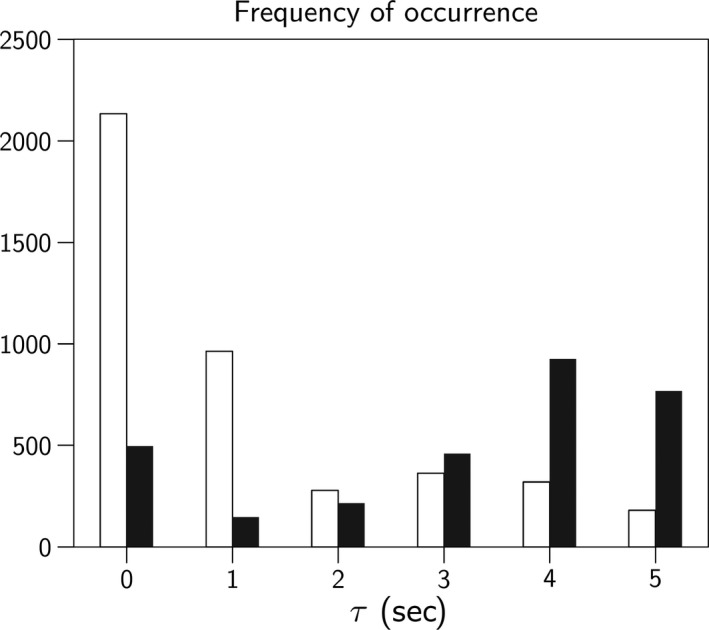
The distributions of occurrences of optimal delay *τ* in baseline condition (white) compared with drug state atropine (black), aggregated for the eight healthy subjects. In baseline, *τ* is clustering around 0 and 1 sec delay, under atropine the remaining reflex tends to sympathetic dominance and τ clusters around 3–5 sec. The probability that these distributions are the same is *P* < 0.001.

Differences in optimal delay *τ* were significant (*P* ≤ 0.001) between study period atropine and atropine + propranolol on the one hand and all others, but not between the two. Atropine administration was associated with a lengthening in the delay to 3 sec (Fig. 6).

### Near‐continuous assessment of baroreflex sensitivity

The total number of determinations of xBRS cumulative over all study periods was more than 28,000, an average of approximately 28 per minute (33/min over all baseline conditions). Except for the atropine sessions, where fewer values were scored, the number of xBRS determinations per minute did not differ significantly. Thus, an xBRS estimate was available on average almost every two seconds, although they were somewhat irregularly distributed over time. Of the distances between successive xBRS determinations, 75% were at distance 1 sec and 12% at 2 sec. Larger distances occurred in the remaining 13%, and a distance of 15 sec occurred at least once per study period. Apparently, baroreflex activity cannot always be detected by this method, in periods of very little variability (as under atropine) or when the reflex is overruled by other neural activity.

When the xBRS distributions were tested, 6 out of 56 were accepted as normal. By contrast, 40 were accepted as log‐normal and the last 10 were either normal or log‐normal, justifying the use of geometric averaging.

### Relation between xBRS and heart rate

**Figure 7:**
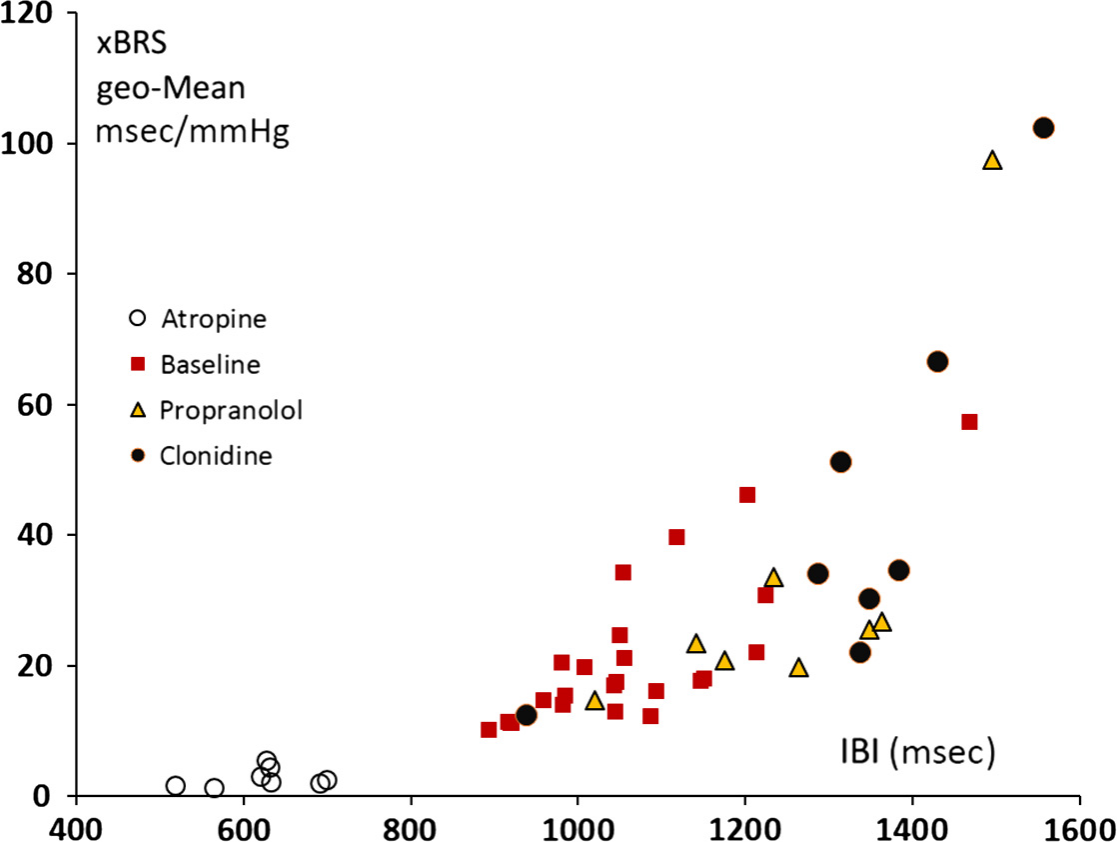
xBRS geometric mean is plotted versus the average interbeat interval (IBI) for all subjects and four different conditions (cf embedded legends) showing proportionality between both parameters. Note the two high values of xBRS around 100 (cf. Fig. 4) which are no longer obvious outliers.

It has repeatedly been argued that many of the usual heart rate variability (HRV) measures can be replaced by looking at heart rate itself (Stauss 2014), since the two are strongly correlated: lower heart rates, more variability. If one considers the baroreflex to be the main underlying factor for HRV (DeBoer et al. 1987), the same should be found here. And indeed, as Figure 7 shows, this also holds for xBRS: longer heart periods go along with higher xBRS. Even the two “sleeping outliers” now seem to fit into the overall pattern.

## Discussion

In this study, the main finding was that cross‐correlation baroreflex sensitivity values are variable around a more or less stable level per study period, while these levels were in agreement with the classical drug‐induced (Smyth et al. 1969), and TG‐BRS method (Parlow et al. 1995). The xBRS variability was proportional to its mean level, gSD being approximately 40% of gM in these young healthy volunteers, independent of the administration of autonomically active drugs. The changes in optimal delay τ are compatible with a cardiac baroreflex under vagal (baseline and clonidine) predominantly vagal (propranolol) or sympathetic (atropine) control. The xBRS technique allows one to look at changes in baroreflex sensitivity and – delay with a time resolution close to 2 sec. This will enable the detection of time patterns in that variability, probing into the activity of the medullary neuron pools which are also actively involved in other homeostatic mechanisms than cardiovascular control, like for instance respiration. Some consideration on whether the xBRS computational algorithm produces reliable numbers as BRS estimates are given in the Appendix.

### Has variability in BRS levels been observed previously?

First, we discuss variability in BRS *as a level*, defined by the circumstances the subject is in. This study showed a range of time‐averaged xBRS of 20 to 1 (Table 2), confirmed by drug injections phenylephrine and nitroprusside. It has been known for some time that BRS obtained with the Oxford technique, within subjects, differs over a range at least as wide as 10 to 1. Smyth et al. (1969), in the first study to use pharmacological pressor responses to estimate BRS, found sensitivities of 6 msec/mmHg in awake, 11 in resting, and 26 msec/mmHg in sleeping subjects, thus varying over a range of a factor of 4.3. Several studies (Abrahamsson et al. 2003) have observed a drop in BRS during physical exercise by a similar factor of 4 or greater. Combined, this yields a 17:1 range from sleep to exercise even without the application of drugs like propranolol. Head‐up tilt reduces BRS by a factor of 2–3 (Westerhof et al. 2006). It thus appears that BRS as a level is highly variable depending on the state a subject is in and xBRS ranges correspond to those found in the literature. In addition, values during sleep are a factor of two higher than during rest (Smyth et al. 1969), as also found in the present study in the two subjects who had fallen asleep (Fig. 4). This high variability of the baroreflex sensitivity is in line with the fact that this physiological mechanism is constantly engaged to buffer systolic pressure changes on a beat‐by‐beat basis. This occurs during sleep, sitting rest or strenuous exercise, with changes in sensitivity according to the condition.

In a cross‐sectional study, Pinna et al. (2000) demonstrated the proportionality between BRS standard deviation and mean level (coefficient of variation) in 454 patients. They had measured BRS three times per patient by the Oxford Phe bolus injection method. However, Figure 4 shows an almost perfect proportionality between standard deviation and mean xBRS with a slope of 0.4, Pinna et al.'s data fill the area between the slope of 0.4 and the slope of 0 (abscissa axis) thus many subjects have a coefficient of variation lower than 0.4. The 40% variability that we find seems an upper limit. An explanation might be that the formerly used study group consisted of older patients with a recent myocardial infarction, whereas our subjects were all young, healthy, supine volunteers.

### Possible origin of variability of instantaneous cardiac baroreflex sensitivity

The comparison of averaged instantaneous xBRS to the “gold standard” of Phe‐ and SNP‐induced transients treats the observed variability in the new signal as an obnoxious side effect which should be discarded by averaging over suitable periods. However, the presence of very low frequency periodicity in the signal may open up a much wider vista on the working of the baroreflex, as part of the homeostatic mechanisms, which find their conductor in the lower brain stem. It is well‐known that respiration, as defined by rate and depth, shows the same periodicities as observed here in xBRS: around 30–50 sec (van den Aardweg and Karemaker 1991, 2002) reason why we hypothesize a causative relation between respiratory and xBRS variability. Due to the lack of a reliable respiration signal in the present data set, we are unable to substantiate this claim; the present experiments were not designed to look into the relation between blood pressure and respiration. Slow oscillations of baroreflex gain have also been observed by Eckberg and Kuusela (2005) and Eckberg et al. (2013), using short‐time Fourier analysis of blood pressure and heart rate recordings. Those authors used a sliding window of (the shortest) 15 sec duration to estimate BRS from the cross‐spectrum between systolic pressure and heart period in the LF band (0.04–0.15 Hz). Shifting the window by 2 sec, they obtained BRS time series with oscillations concentrated in the VLF band (around 80 sec, as reported in their 2005 study) or in the LF band (around 18 sec in the 2013 study). The oscillations reported in these studies have therefore periods in one case longer, in the other case shorter than the ones we observed (20–50 sec), but as already mentioned, there is much variability in these slow rhythms. More problematic are the constraints, which one incurs by using Fourier techniques to compute near‐instantaneous BRS. The xBRS method employed here appears a better choice, since it does not suffer such rigid constraints, which, strictly speaking, were not met in the Eckberg/Kuusela studies. The highest frequency to be observed by xBRS, if one considers the numbers only independent if there is no overlap in the data points, is 1/(2*10) = 0.05 Hz. A close inspection of the applied signal analysis techniques has practical consequences: if the analysis method tends to give answers skewed toward very low or ultralow frequencies, one would start looking for other than neuronal mechanisms for explanations, like levels of circulating angiotensin, which turned out not to be the culprit (Eckberg and Kuusela 2005). In our observations, the baroreflex sensitivity oscillations span a range of frequencies, which we consider compatible with oscillations due to respiratory control. Respiration is but one of the many mechanisms due to which the baroreflex is continually changing its sensitivity, all the while adapting itself to the immediate requirements of the organism and allowing blood pressure and heart rate to change accordingly (Wesseling and Settels 1985; Di Rienzo et al. 1995; Wesseling et al. 1995; Karemaker and Wesseling 2008).

### Perspective

The xBRS method seems a promising tool to catch a number of cardiac control issues at the same time: for one, the cardiac baroreflex, with good sensitivity and with a time resolution of only a few seconds and comparable with the tangent method (Parlow et al. 1995) for BRS values. Simultaneously, optimal delay *τ*, either short or long, respectively, indicates vagal versus sympathetic dominance acting in the cardiac baroreflex. And finally, it can provide information on the condition of the central nervous system when we concentrate on its variability. In that respect, the method can prove useful when tracking transient changes in BRS during sleep, in exacting conditions (exercise, altitude, daily practice e.g., critical care/anesthesia), or during clinical physiology interventions (standing up and tilt responses (Westerhof et al. 2006)). Clearly, no analysis method can pretend, or needs, to cover all the aspects of the cardiac baroreflex with minute precision. Rather, the issue is to combine various tools to approach as closely as possible physiology or pathophysiology.

## Conclusion

With the introduction of the xBRS method, we have found a way to momentarily look into one of the most important mechanisms controlling the cardiovascular system: the cardiac baroreflex. All it takes is a finger blood pressure recording, whereby the exact level of blood pressure is of less consequence than the reliable tracking of moment‐to‐moment changes. In that respect, it is a promising new tool to add to monitoring devices, giving the opportunity to track the autonomic control and judge the patient's status on a near‐continuous timescale.

## Note explaining the history of this paper

The first version of this paper has been conceived by the first author, the late Prof. K. H. Wesseling. Due to his disease and untimely demise (Westerhof et al. 2015), the draft paper has been “floating around” for a considerable time. The coauthors, finally, decided to revise the paper and to adapt it to recent literature.

## Conflict of Interest

The authors declare no conflict of interest.

## Note

1 To calculate the back‐transformed gSD, we used the formula: gSD = gM*√(exp(SDln^2^)−1) where SDln is the standard deviation of the log_e_ transformed distribution (see https://en.wikipedia.org/wiki/Log-normal_distribution).

## Is the xBRS computational algorithm producing noisy numbers as BRS estimates?

Take a section of continuous blood pressure recording and list the sequence of systolic blood pressures. The systolic values are not constant but fluctuate in time. The fluctuations may be small or large, sinusoidal or complex. In spectral analysis of the fluctuations, almost always two peaks stand out, one near 0.1 Hz (the so‐called 10 sec rhythm), and one near 0.25 Hz, synchronous with respiration. These peaks are not narrow like in a line spectrum but broad and smeared out as is typical for amplitude and frequency modulated sinusoids. In addition, there is often a band visible at frequencies below 0.1 Hz, due to respiratory variability (van den Aardweg and Karemaker 1991). This band is often buried in the overall 1/*f* noise that is present in the systolic pressure records of longer duration such as 24 h (Di Rienzo et al. 1995). A record of interbeat intervals shows similar fluctuations and spectral components.

Looking at short (20 min) sections of simultaneous systolic pressure and interbeat interval fluctuations one may thus expect to see a 0.1 Hz sinusoid of which the amplitude and frequency are modulated. In addition, there is some noise superimposed and possibly a baseline drift. With these signals, we calculate the ratio of the interbeat interval fluctuation amplitude (expressed as standard deviation) with the standard deviation in systolic pressure. This ratio we call baroreflex sensitivity if the spontaneous fluctuations are coherent and interval fluctuations follow pressure fluctuations. Every second, the xBRS algorithm checks if coherent waves are present in the 10‐sec wide window of blood pressure and interbeat interval data.

The 10 sec rhythm is “waxing and waning” and is sometimes completely absent for short periods (Golenhofen and Hildebrandt 1958). Computation of baroreflex sensitivity is only possible when there is beat‐to‐beat variability in blood pressure and interbeat interval. For xBRS to work optimally, the 10 sec rhythm should be continuously present, not obscured by interfering activity outside the blood pressure – heart rate loop. The present outcome: one measurement on average per 2 sec is probably the best obtainable result. This is many times more than yielded by the standard “sequences” technique (Di Rienzo et al. 1985). How is this possible? Consider a 10‐sec window in the pressure and interval data stream, and plot interval versus pressure. Compute the correlation between pressure and interval and repeat this for the interval delayed with respect to pressure by 1, 2…5 sec. Select the one delay giving the highest positive cross‐correlation. Normally, in young adults the optimal delay τ will be 0 or 1 sec (Karemaker 1980; Karemaker and DeBoer 2017), but it may be 2 or 3 sec depending on the degree of vagal versus sympathetic dominance. Not imposing a minimum change in pressure and interbeat interval, as is usual in the sequences technique, and choosing the highest cross‐correlation may lead to more “hits” than by just imposing a delay of 0 or 1 sec (Di Rienzo et al. 1985). Thus, more correlations will be significant and produce a result in the output file. In addition, the xBRS technique looks at all data in the 10 sec window. It does not specifically look for just up‐ or down going sequences, but the pressure and interval signals may go up and down in the same window as long as they show (positive) correlation. In the present study, this correlation was required to be significant at the *P* < 0.05 level. However, even when this requirement is relaxed as suggested earlier (Bernardi et al. 2010; Porta et al. 2013) and ultimately the correlation is just positive, not even significant, then still do the numbers not change importantly: the average xBRS remains the same, so does the coefficient of variation (geometric SD/geometric mean). The number of detected xBRS estimates increases to 90% of the time in the baseline recordings and under clonidine or propranolol, less for atropine blockade or atropine + propranolol blockade. However, since the combination of atropine + propranolol at the applied dosages is supposed to block all muscarinic and ß‐adrenergic effects on heart rate, any remaining BRS must be due to insufficient blockade, or to transmitters which are not blocked by atropine or propranolol, like Vaso Intestinal Peptide, one of the known co‐transmitters of acetylcholine in the autonomic nervous system (Roossien et al. 1997; Karemaker 2017) or to direct or indirect mechanical effects on blood pressure and the sinoatrial node, for instance, by respiration, as these may be produced by the complex interactions in the ganglionated cardiac plexuses (Armour 2004).

Can the mean xBRS levels as present in Figure 2 be trusted as true BRS values for the study period? In Figure 1, it can be seen that scatter is present between the xBRS values and the nonsimultaneous tangent TG BRS, as has been noted by Watkins et al. (1996), Pinna et al. (2000) and Lipman et al. (2003) and others. Clearly, however, the scatter between the Phe and the SNP responses on the one hand and the tangent values on the other also show scatter, even though the Phe and SNP responses together constitute the input to the sigmoid from which the tangent is derived. Note that the correspondence between xBRS and TG BRS per study period as presented in Figure 2 has less scatter when averaged over the whole group. Thus, more averaging further reduces the scatter. A limitation to the accuracy obtained in this study is that one cannot expect values obtained from two different methods to be identical unless they are at least completely simultaneously taken over the same time span, which is not the case here. In addition, a method should not disturb the baroreflex itself. Using spontaneous oscillations in a method such as xBRS obviously does not disturb the baroreflex but this is not the case for drug induced baroreflex responses. Finally, spontaneous fluctuations also occur during the drug‐induced responses, corrupting the sigmoids and adding scatter.
